# EEG-based human emotion recognition using entropy as a feature extraction measure

**DOI:** 10.1186/s40708-021-00141-5

**Published:** 2021-10-05

**Authors:** Pragati Patel, Raghunandan R, Ramesh Naidu Annavarapu

**Affiliations:** Department of Physics, School of Physical, Chemical, and Applied Sciences, Pondicherry University, Puducherry, 605014 India

**Keywords:** EEG, Emotion recognition, Entropy measure, Feature extraction, Signal processing

## Abstract

Many studies on brain–computer interface (BCI) have sought to understand the emotional state of the user to provide a reliable link between humans and machines. Advanced neuroimaging methods like electroencephalography (EEG) have enabled us to replicate and understand a wide range of human emotions more precisely. This physiological signal, i.e., EEG-based method is in stark comparison to traditional non-physiological signal-based methods and has been shown to perform better. EEG closely measures the electrical activities of the brain (a nonlinear system) and hence entropy proves to be an efficient feature in extracting meaningful information from raw brain waves. This review aims to give a brief summary of various entropy-based methods used for emotion classification hence providing insights into EEG-based emotion recognition. This study also reviews the current and future trends and discusses how emotion identification using entropy as a measure to extract features, can accomplish enhanced identification when using EEG signal.

## Introduction

Emotions are biological states related to the nervous systems, which are usually reflection of changes in neuro-physiological condition. Being an indispensable part of human life, if emotions could be anticipated by machines precisely, it would accelerate the progress of artificial intelligence or the brain–computer interface field [1]. Presently there is no scientific agreement on a definition of emotions. One of the definitions, as given by William James, claims that "the bodily changes follow directly the perception of the exciting fact, and that our feelings of the changes as they occur are emotion” [2]. As an evolving field of research with vital importance and vast implementation, emotion classification has drawn interest from different disciplines like neuroscience, neural engineering, psychology, computer science, mathematics, biology, physics and has always remained in the spotlight [3, 4]. Various experiments are being done to accomplish higher instinctive human–computer interaction and ultimate goal is to devise advanced gadgets, which would distinguish various human emotions, in real-time [5]. With the absence of the capability to quantify emotions, computers and robots cannot naturally connect with humans. Therefore, human emotion recognition is the key technology for human–machine interaction [6, 7].

### Why EEG for emotion recognition?

In current practice, emotion recognition is done widely in two ways, either by making use of non-physiological signals or by making use of physiological signals. Non-physiological methods use text, speech, facial expressions, or gestures. Most of the previous studies are based on this method [8–10]. However, this method cannot be considered reliable, as facial gesture or voice tone can be voluntarily obscured. Unlike the first, the second approach employing physiological signals seems more efficient and reliable because one cannot restrain them willfully [3]. At present among all available physiological signals, emotion detection using the EEG signal has become most popular non-invasive one as EEG efficiently records the electrical activity of brain [11]. With the advancement of sensor networks [12, 13], intelligent sensing system [14–16], and energy-efficient biomedical systems [17, 18], EEG-based methods has gained feasibility. Additionally, several researches have proved the reliability of EEG for application in the BCI [19], electronic gadgets [20, 21] as well as in medicine to closely inspect various brain disorders [22–24]. Some EEG-based research indicate that EEG builds enhanced databases in comparison with other available emotion databases like the non-physiological datasets. Thus because EEG is meticulously tied in with brain activities [25–27] and also because it is immediate and comparatively reliable than electrodermal activity (EDA; sometimes known as galvanic skin response, or GSR), electrocardiogram (ECG), photoplethysmography (PPG), electromyography (EMG), etc., is highly recommended over any other physiological signals [28, 29].

### How does entropy contribute in emotion recognition?

Entropy is a nonlinear thermodynamic quantity that specifies the system’s degree of randomness. Measures of entropy are usually employed to assess the inconsistency, intricacy and unpredictability of biomedical data sets and this principle of entropy has also been extended to study the complexity of EEG signals. A number of studies are available to prove that entropy measures have a significant capability to access knowledge regarding regularity from EEG data sets [18]. Therefore, entropy, being a nonlinear feature that measures the level of randomness of any system, is effective in distinguishing various emotions based on their level of irregularity in the EEG signal.

### Major contributions of present study

In short, contributions of this article are:

1. From analysis, it has been discovered that recurrence quantification analysis entropy along with ANN-based classifiers approach dominates other approaches.
2. This analysis will assist researchers to determine the combination of entropy characteristics and classification methods that is more appropriate for further enhancement of the current emotion detection methods.
3. This will assist learners in better comprehension of various existing EEG datasets of emotions.
4. Study also proposes that entropy function algorithms can be effective, for other information-retrieval tasks such as detection of emotion-related mental disorders, in addition to emotion recognition.
5. Finally, through the present review and analysis, certain findings and suggestions have been listed for further studies in this field.

**Fig. 1:**
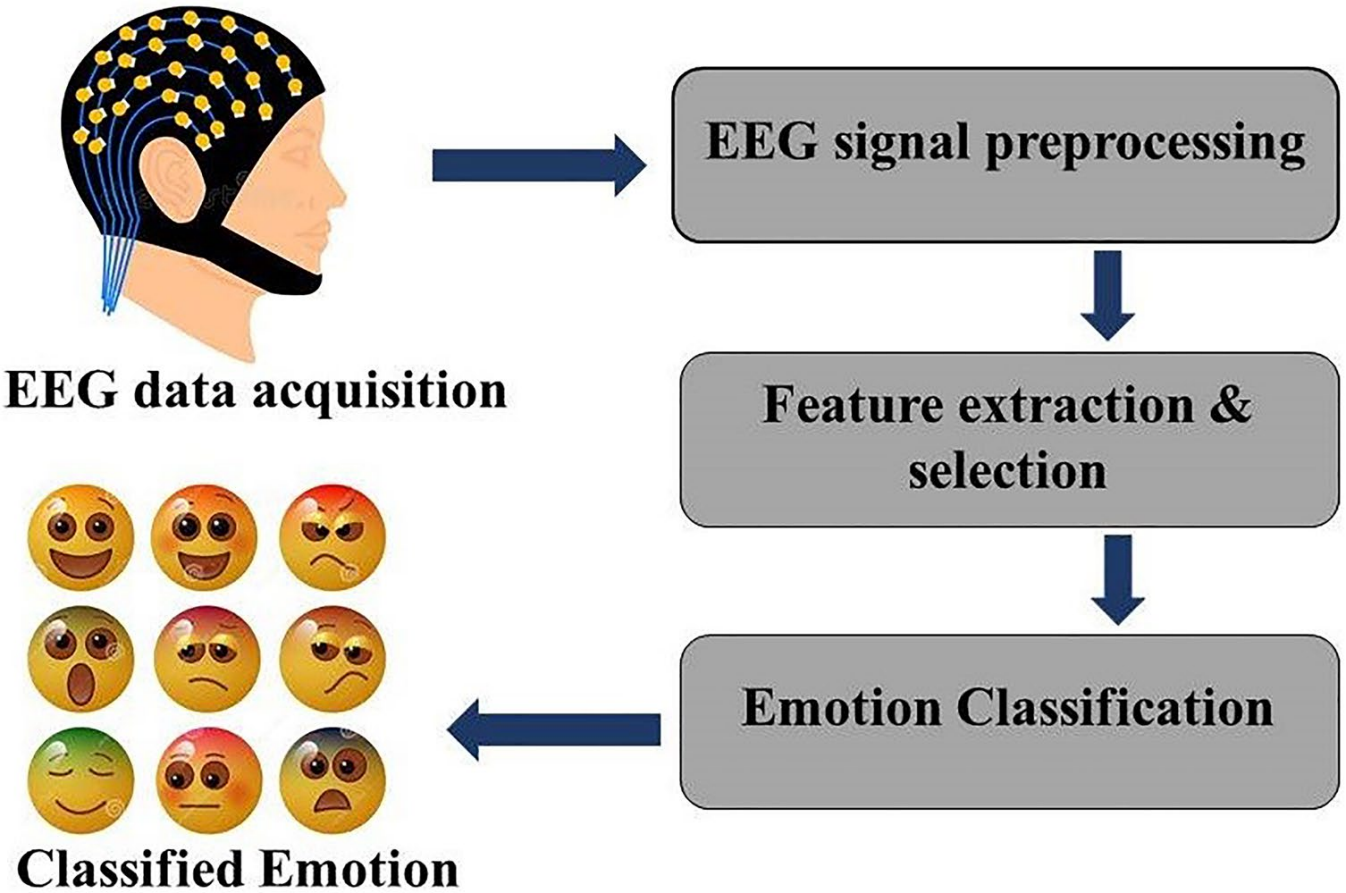
Generalized block diagram of the proposed methodology

A generalized block diagram of the proposed methodology in various papers discussed in this review is given in Fig. 1. The methodology includes four main tasks; first is the data acquisition, i.e., EEG device records a high-quality signal from the brain. These raw signals are often contaminated with unwanted noise and artifacts. The second task is the preprocessing of the raw signal to remove or minimize these noise and artifacts, which uses different filters, and then the raw signal is down-sampled to some sampling frequency. After the signal is preprocessed, feature extraction is carried out. Here various entropy methods are used to extract significant information from the EEG data. Last is the classification task: after selecting the features useful to the psychological state, classifiers must be trained in such a way that different emotional states can be categorized using the extracted features.

There is increasing evidence that entropy-based methods can provide higher accuracy for emotion recognition [29–33]. The objective of this paper is to comprehend various entropy measures for EEG-based emotion classification. The article is structured as follows: Sect. 2 presents the theories of emotion and EEG as psycho-physiological measures for emotion evaluation. Section 3 discusses the implementation of EEG measures for emotion recognition. Section 4 provides an analysis of the previous research works on EEG-based emotion recognition using different entropy measures. Section 5 concludes with the findings of this review on emotion recognition and also provides some suggestions for future research in this field.

## Theory and measures of emotion

### Theories of emotion

**Fig. 2:**
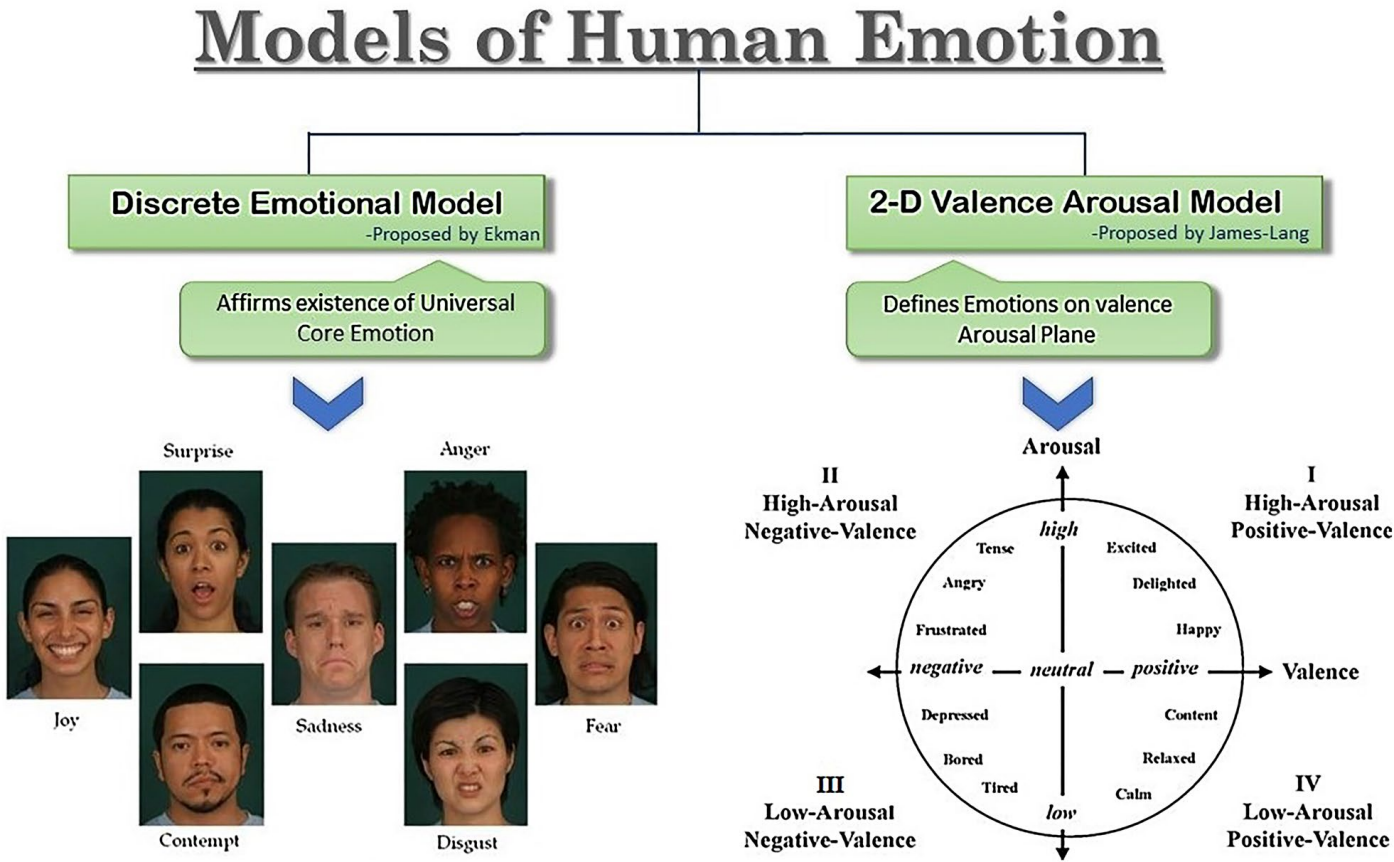
Models of human emotion widely used in emotion recognition

Emotions show up in our day to day lives and they influence our human awareness significantly [34]. Principally, an emotion is a psychological state that arises unconsciously instead of a conscious effort and it appears as physiological changes in the body [35]. Different theories have been proposed by psychologists and neuroscientists regarding why and how any living body experiences emotion [36]. However, the two widely used models in emotion recognition are the discrete emotional model and the two-dimensional valence-arousal model, proposed by Ekman [37] and Lang [38], respectively. The discrete emotional model affirms the existence of a few primitive or core emotions universally in all perceptions. Different psychologists have proposed various classes of emotions, but a substantial agreement lies for six emotions specified as happiness, sadness, surprise, anger, disgust, and fear [39]. The dimensional model attempts to conceptualize human emotions by defining where they lie in the two or three dimensions [36] and classifies emotions depending on their valence-arousal scale. Valence indicates pleasantness and ranges from negative to positive. Arousal represents the activation level and ranges from low to high [40]. Figure 2 summarizes the models of emotions mentioned above.

### EEG as a psycho-physiological emotion assessment measure

Psycho-physiology refers to the part of brain science that deals with the physiological bases of psychological processes. Regardless of whether an individual expresses the emotion through speech and gesture, a change in cognitive mode is unavoidable and measurable [41] as receptive nerves of the autonomic nervous system are stimulated once an individual is positively or negatively excited. This stimulation elevates fluctuation in heart rate, increases respiration rate, raises blood pressure, and decreases heart rate variability [42].

Monitoring brain activity through EEG is among the most widely accepted psycho-physiological measures used in the research field of the BCI. EEG databases correspond to the functions of the central nervous system (CNS) which monitors and records activity in the brain.

## Methods and materials

### Emotion elicitation stimuli

Emotion elicitation is required in the subject to obtain a high-quality database for building an emotion classification model [6, 43]. To obtain a good emotion dataset it is important to elicit the emotion in the subject naturally as EEG is measured in ANS/CNS. Many different protocols for emotion elicitation on subjects have been proposed using various types of stimuli. The most common of all is audiovisual. Images have also been used to elicit emotions, international affective picture system (IAPS) [44, 45] is a very well-known project to develop an international framework for emotional elicitation based on a static picture. The link between music and emotions has also inspired the use of music and audio stimuli to elicit emotions [40]. Memory recalling [43] and multimodal approach [6] are some other strategies used.

The purpose of the emotion elicitation technique is to stimulate the desired emotion in the subject by eliminating the possibility of stimulating multiple emotions. A study by Gross and Levenson reveals that psychologically characterized movies attained better outcomes considering its dynamic profile [46].

### Data used

In this review article, results from various entropy approaches have been reviewed. Different researchers used different emotion database as per the need and suitability of the data for better results.

There are two benchmark databases; dataset for emotion analysis using physiological signals (DEAP) and SJTU Emotion EEG Dataset (SEED) that are widely used. The other datasets were similarly recorded, but are not publicly available for use.

#### DEAP dataset

Dataset for emotion analysis using physiological signals (DEAP) is a multimodal dataset designed to study human cognitive states by Queen Mary University in London. The database has been obtained using a BioSemi acquisition system of 32 channels and it recorded the electroencephalogram (EEG) and peripheral physiological signals of 32 participants as each subject viewed 40 1-min music video excerpts. The subjects scored the videos according to the extent of arousal, valence, like/dislike, dominance, and familiarity. Frontal face video was also tracked for 22 of the 32 subjects. A unique stimulus selection approach has been used, using affective tag retrieval from the last.fm website, video highlight identification, and an online assessment tool. The dataset is made freely accessible, and it allows other researchers to use it to check their methods of estimating the effective state. The dataset was first presented in a paper by Koelstra et al. [46]. For further specifics on DEAP database, please find the details online at https://www.eecs.qmul.ac.uk/mmv/datasets/deap/index.html.

#### SEED dataset

The SEED dataset includes 62-channel EEG recordings from 15 subjects (7 males and 8 females, 23.27 $\pm$ 2.37 years) according to the international standard 10–20 system. The emotions of the subjects are triggered through 15 video shots, and every video shot is of 4 min duration. It measures three types of emotions (positive, neutral, negative) and every category of emotion is associated with five video shots, respectively. At a time interval of 1 week or longer between multiple sessions, each participant was advised to enroll in the experiments for three sessions [47, 48]. For further specifics on SEED database, please find the details online at http://bcmi.sjtu.edu.cn/home/seed/seed.html.

### Entropy feature extraction

In building an emotion recognition system, different features are required to be retrieved, whichever better describes the behavior (either static or dynamic) of brain electrical activity during different emotional states. Distinct kinds of emotions depending on different entropy characteristics are assessed in this article.

Entropy function is a dynamic feature that describes the chaotic nature of any system and evaluates the amount of information acquisition which could be employed to isolate necessary information from the interfering data [49]. Greater the value of entropy, greater is the irregularity of the system. This section provides a concise description of various entropy used as feature to classify different emotions.

#### Sample entropy

Sample entropy (SampEn) quantifies a physiological signal’s complexity irrespective of the signal length and therefore has a trouble-free implementation. Conceptually, sample entropy is based on the conditional probability that two sequences of length ‘*n* + 1’ randomly selected from a signal will match, given that they match for the first ‘*n*’ elements of the sequences. Here ‘match’ means that the distance between the sequences is less than some criterion ‘*k*’ which is usually 20% of the standard deviation of the data sequence taken into account. Distance is measured in a vector sense. Defining ‘*k*’ as a fraction of the standard deviation eliminates the dependence of SampEn on signal amplitude. The conditional probability is estimated as the ratio of the unconditional probabilities of the sequences matching for lengths ‘*n* + 1’ and ‘*n*’, and SampEn is calculated as the negative logarithm of this conditional probability [49]. Thus, SampEn is defined as:

1 $${\text{Sample}}\;{\text{Entropy}} = - \log \left( {\frac{{A^{n} \left( k \right)}}{{B^{n} \left( k \right)}}} \right),$$

where $B^{n} (k)$ is the estimated probability that two sequences match for n points, and $A^{n} \left( k \right)$ is the estimated probability that the sequences match for *n* + 1 points. $A^{n} \left( k \right)$ and $B^{n} (k)$ are evaluated from data using a relative frequency approach.

Sample entropy is comparatively reliable and decreases the bias of approximate entropy [31]. Greater value of estimated sample entropy suggests that signal is extremely unpredictable and lower value suggests that signal is predictable. The value of ‘*n*’ has to be considered as per the preference of the researcher and it differs from work to work.

SampEn’s advantage is that it can distinguish a number of systems from one another. It gives a much better result than approximate entropy with the theory of random numbers. Self matches are not included in this entropy so the bias decreases. The entropy esteems are relatively steady across various sample lengths. However, lack of consistency for sparse data is the key downside of SampEn.

#### Dynamic sample entropy

As the name suggests dynamic sample entropy (DySampEn) is the dynamic extension of the Sample entropy and is applied to evaluate the EEG signal. DySampEn is often seen as the dynamic feature of EEG signals that can track the temporal dynamics of the emotional state over time. Dynamic sample entropy determination strategy follows the calculation of SampEn from EEG signals by sliding time windows (a set of consecutive time windows) [29] by employing sliding time windows with window length $t_{w}$ and moving window length ∆*t*, the DySampEn can be expressed as:

2 $${\text{DySampEn}}\left( {n,k} \right) _{\left\langle l \right\rangle } = {\text{SampEn}}\left( {n,k} \right)_{\left\langle l \right\rangle } , \quad 1 \le l \le w,$$

where subscript $\left\langle l \right\rangle$ represents sliding time windows (*k* = 1,2,3,…$w$) and $w = \left[ {\frac{{T - t_{w} }}{\Delta t}} \right] + 1$ is measure of sliding time windows and T is the total length of EEG signal and $\left[ \cdot \right]$ corresponds to floor function that rounds $\frac{{T - t_{w} }}{\Delta t}$ to largest integer not exceeding $\frac{{T - t_{w} }}{\Delta t}$.

Time window length $t_{w}$ and moving window length ∆*t* have to be taken as per research need. As EEG features a temporal profile, the benefits of this entropy is that it can provide more accurate emotionally relevant signal patterns for emotion classification than sample entropy [18, 29].

#### Differential entropy

Differential entropy (DE) is the entropy of a continuous random variable and measures its uncertainty. It is also related to minimum description length. One may describe the mathematical formulation as

3 $$h\left( X \right) = - \int \limits_{X}^{{}} f\left( x \right) \log \left[ {f\left( x \right)} \right]{\text{d}}x,$$

where *X* is a random variable, $f\left( x \right)$ is its probability density. So for any time series *X* obeying gauss distribution $N\left( {\mu ,\sigma^{2} } \right)$, its differential entropy can be expressed as [50]:

4 $$\begin{aligned} h\left( X \right) & = - \int \limits_{ - \infty }^{\infty } \begin{array}{*{20}c} {\frac{1}{{\sqrt {2\pi \sigma^{2} } }}e^{{ - \left[ {\frac{{\left( {x - \mu } \right)^{2} }}{{2\sigma^{2} }}} \right] }} } \\ \end{array} \log \left[ {\frac{1}{{\sqrt {2\pi \sigma^{2} } }} e^{{ - \left[ {\frac{{\left( {x - \mu } \right)^{2} }}{{2\sigma^{2} }}} \right]}} } \right]{\text{d}}x \\ & = \frac{1}{2} \log \left( {2\pi e\sigma^{2} } \right). \\ \end{aligned}$$

Disadvantage is estimation of this entropy is quite difficult in practice, as it requires estimation of density of *X*, which is recognized to be both theoretically difficult and computational demanding [3, 51].

#### Power spectral entropy

Power spectral entropy (PSE) is standardized model of Shannon’s entropy. It utilizes the component of the power spectrum amplitude of the time series to evaluate entropy from data [3, 50], i.e., it measures the spectral complexity of any EEG signal and so also regarded as frequency domain information entropy [51].

Mathematically it is given by

5 $${\text{PSE}} = - \mathop \sum \limits_{f} P_{f} \log P_{f} ,$$

where $P_{f}$ is power spectral density.

**Shannon’s entropy (ShEn)** is a set of relational variables that changes linearly with the logarithm of a range of possibilities. This is also a data spread metric, which is widely applicable in a system’s dynamic order determination.

Shannon’s entropy being based on the additivity law of the composite system, i.e., if a system is divided into two statistically independent subsystems *A* and *B* then as per additivity law

$$S\left( {A, \, B} \right) = S\left( A \right) + S\left( B \right),$$

where *S* (*A*, *B*) is the total entropy of the system and *S*(*A*) and *S*(*B*) are entropy of subsystems *A* and *B*, respectively. So, Shannon’s entropy successfully addresses extensive (additive) systems involving short-ranged effective microscopic interactions. Now, physically ‘‘dividing the total system into subsystems’’ implies that the subsystems are spatially separated in such a way that there is no residual interaction or correlation. If the system is governed by a long-range interaction, the statistical independence can never be realized by any spatial separation since the influence of the interaction persists at all distances and therefore correlation always exists for such systems. This explains the disadvantage of ShEn that it fails miserably for non-extensive (non-additive) systems that is governed by long-range interactions. It overestimates the entropy level when a larger number of domains are considered, and also does not clarify the temporal connection between different values extracted from a time series signal [18].

#### Wavelet entropy

One of the quantitative measures in the study of brain dynamics is wavelet entropy (WE). It quantifies the degrees of disorder related to any multi-frequency signal response [52].

It is obtained as

6 $${\text{WE}} = - \mathop \sum \limits_{i < 0} P_{i} \ln P_{i} ,$$

where $P_{i}$ defines the probability distribution of a time series signal and $i$ defines different resolution level [18].

It is utilized to recognize episodic behavior in EEG signal and provides better results for time-varying EEG [53]. The benefit of wavelet entropy is it efficiently detects the subtle variations in any dynamic signal. It takes lesser computation time, noise can be eliminated easily and its performance is independent of any parameters [18].

#### EMD approximate entropy

Approximate entropy (ApEn) is a ‘regularity statistics’ measuring the randomness of the fluctuation in given data set. Generally, one may assume that existence of repeated fluctuation patterns in any data set makes it a little less complex than other dataset without many repetitive patterns. Approximate entropy ensures identical patterns of predictions are not accompanied by further identical patterns. A data set with a lot of recurring motifs/patterns has notably lower ApEn, whereas a more complex, i.e., less predictable data set has higher ApEn [54]. ApEn estimation algorithm is described in many papers [33, 54–57]. Mathematically it can be calculated as

7 $${\text{ApEn}}\; \left( {n,k,N} \right) = \ln \left( {\frac{{C_{n} \left( k \right)}}{{C_{n + 1} \left( k \right)}}} \right),$$

where $C_{n} \left( k \right)$ and $C_{n + 1} \left( k \right)$ are pattern mean of length $(n)$ and $\left( {n + 1} \right)$, respectively. ApEn is robust to noise and relies on a less amount of data. It detects changes in series and compares the similarity of samples by pattern length $n$ and similarity coefficient ‘$k$’. The appropriate selection of parameters ‘*n*’ (subseries length), *k* (similarity tolerance/coefficient) and *N* (data length) is critical and are chosen as per research needs. Traditionally, for some of clinical datasets, ‘*n*’ is to be set at 2 or 3, ‘*k*’ is to be set between 0.1 and 0.25 times the standard deviation of time series taken into account to eliminate the dependence of entropy on signal’s amplitude and *N* as equal to or greater than 1000. However, these values do not always produce optimal results for all types of data. The paper cited presents a method that employs the empirical mode decomposition (EMD) to disintegrate the EEG data and then calculates ApEn of disintegrated data and so, is called E-ApEn. EMD is a time frequency analysis method that decomposes nonlinear signals into oscillations at various frequencies.

The advantages of EMD-ApEn are that it is measurable for shorter datasets with high interference and it effectively distinguishes various systems based on their level of periodicity and chaos [49, 54]. The disadvantages are it strongly depends on the length of input signal [58]. Meaningful interpretation of entropy is compromised by significant noise. As it depends on length, it’s a biased statistics [18, 59].

#### Kolmogorov Sinai entropy

The volatility of data signal over time is assessed using entropy defined by Kolmogorov Sinai shortly known as KS entropy. It is determined by identifying points on the trajectory in phase space that is similar to each other and not correlated with time. Divergence rate of these point pairs yields the value of KSE [60], calculated as

8 $${\text{KSE}} = \mathop {\lim }\limits_{r \to 0} \mathop {\lim }\limits_{m \to \infty } \frac{1}{\tau }\frac{{C_{m} \left( {r, N_{m} } \right)}}{{C_{m + 1} \left( {r, N_{m + 1} } \right)}},$$

where $C_{m} \left( {r, N_{m} } \right)$ is the correlation function which provides probability of two points being closer to each other than *r*. Higher KSE value signifies higher unpredictability. Hence KSE does not give the accurate results for signals with slightest noise.

The advantages is that it differentiates between periodic and chaotic systems effectively [61, 62]. And this being decayed towards zero with increasing length is its main limitation [63].

#### Permutation entropy

It is also possible to measure the intricacy of brain activity using the symbolic dynamic theory where a data set is being plotted to a symbolic sequence through which the permutation entropy (PE) is generated. The highest value of PE is 1, signifying that the data series is purely unpredictable; whereas the lowest value of PE is 0, signifying that the data series is entirely predictable [4, 76]. At higher frequency, permutation entropy amplifies with the incongruity of data series while permutations related to reported oscillations are seldom at a lower frequency.

Mathematically PE is described as

9 $${\text{PE}} = - \mathop \sum \limits_{i = 1}^{n} P_{i} \log_{2} P_{i} ,$$

where $P_{i}$ represents the relative frequency of possible sequence patterns, $n$ implies permutation order of $n \ge 2$ [63–65].

Permutation entropy is a measure of chaotic and non-stationary time series signal in the presence of dynamical noise. This algorithm is reliable, effective, and yields instant outcomes regardless of the noise level in data [64, 65]. Thus, it can be used for processing of huge data sets without preprocessing of data and fine-tuning of complexity parameters [13]. The advantages of this entropy are it is simple, robust and less prone to computational complexity. It is applicable to real and noisy data [66], does not require any model assumption and is suitable for the analysis of nonlinear processes [67]. The main limitation is its inability to include all ordinal patterns or permutations of order ‘*n*’, when ‘*n*’ is assigned a larger value for a finite input time series [18, 68].

#### Singular spectrum entropy

Entropy calculated from singular spectrum analysis (SSA) components are known as singular spectrum entropy. SSA is an important signal decomposition method based on principal component analysis, which can decompose the original time series into the sum of a small number of interpretable components. SSA usually involves two complementary stages, one is the stage of decomposition and the other is the stage of reconstruction. The stage of decomposition consists of two steps: embedding and singular value decomposition (SVD). The stage of reconstruction also consists of two steps: grouping and diagonal averaging [69]. Singular spectrum entropy function represents instability of energy distribution and is a predictor of event-related desynchronization (ERD) and event-related synchronization (ERS) [70].

#### Multiscale fuzzy entropy

The measures of fuzziness are known as fuzzy information measures and the measure of a quantity of fuzzy information gained from a fuzzy set or fuzzy system is known as fuzzy entropy. No probabilistic concept is needed to define fuzzy entropy like the other classical entropy that needs probabilistic concept. This is due to the fact that fuzzy entropy contains vagueness and ambiguity uncertainties, while Shannon entropy contains the randomness uncertainty (probabilistic).

Multiscale fuzzy entropy extracts multiple scales of original time series with a coarse-gaining method and then calculates the entropy of each scale separately. Assuming an EEG signal with ‘N-point’ samples is reconstructed to obtain a set of ‘*m*-dimensional’ vectors with $n,$ $r$ and $D_{ij}^{m}$ taken as width, gradient and the similarity degree of the two vectors (fuzzy membership matrix), respectively, final expression for fuzzy entropy appears as

10 $${\text{Fuzzy}}\;{\text{entropy}} \left( {m,n,r} \right) = \mathop {\lim }\limits_{N \to \infty } \left[ {\ln \varphi^{m} \left( {n,r} \right) - \ln \varphi^{m + 1} \left( {n,r} \right) } \right].$$

It can also be defined as $\left[ {\ln \varphi^{m} \left( {n,r} \right) - \ln \varphi^{m + 1} \left( {n,r} \right)} \right]$ for EEG signals where number of given time series sample *N* is limited, where $\varphi^{m} \left( {n,r} \right)$ is a function defined to construct a set of (*m* + 1)-dimensional vector and is taken as:

11 $$\varphi \left( {n,r} \right) = \frac{1}{{\left( {N - m} \right)}}\mathop \sum \limits_{i = 1}^{N - m} \left[ {\frac{1}{N - m - 1}\mathop \sum \limits_{j = 1,j \ne 1}^{N - m} D_{ij}^{m} } \right],$$

with fuzzy membership matrix $D_{ij}^{m} = \mu \left( {D_{ij}^{m} , n , r} \right) = \exp \left( { - (D_{ij}^{m} )^{n} /r} \right).$

For detailed mathematical formulation one can refer to [71, 72]. The advantage of this entropy is that it is insensitive to noise; and is highly sensitive to changes in the content of information [32, 68, 71].

#### Recurrence quantification analysis entropy

This is a measure of the average information contained in the line segment distribution or line structures in a recurrence plot. Recurrence plot is a visualization or a graph of a square matrix built from the input time series. This is one of the state-space trajectories-based approaches of recurrence quantification analysis (RQA). This helps to compute the number and duration of the recurrence of a chaotic system [73]. RQA evaluates the forbidden precession of a data set and is computed to portray a time-varying input signal in contexts of its intricacy and randomness. Recurrence entropy helps detect chaos–chaos transitions, unstable periodic orbits, time delays, and extracts appropriate information from short and nonlinear input signals [18, 30].

### Classification

After extracting features that seem to be appropriate to the emotional responses, these are then used to build a classification model with the intent to recognize specific emotions employing proposed attributes. Different classifiers like K-Nearest Neighbor (KNN) [74], Support Vector Machines (SVM) [3, 29, 32, 51, 52], integration of deep belief network and SVM (DBN-SVM) [75], channel frequency convolutional neural network (CFCNN) [76], multilayer perception, time-delay neural network, probabilistic neural network (PNN) [77], least-square SVM, etc., are used by various researchers for emotion recognition. It is difficult to compare the different classification algorithms, as different research works employ different datasets, that differs significantly in the manner emotions are evoked. In general, the recognition rate is significantly greater when various physiological signals such as EEG, GSR, PPG, etc., are employed together compared to the use of a single physiological signal for emotion recognition [78].

## Discussion

In earlier days, numerous approaches were sought to measure human emotion. Several scientific researches on emotion classification were carried out using the EEG signal in the last couple of years. The human emotion recognition study began with a subject-dependent approach and is now moved more towards a subject-independent approach.

**Fig. 3:**
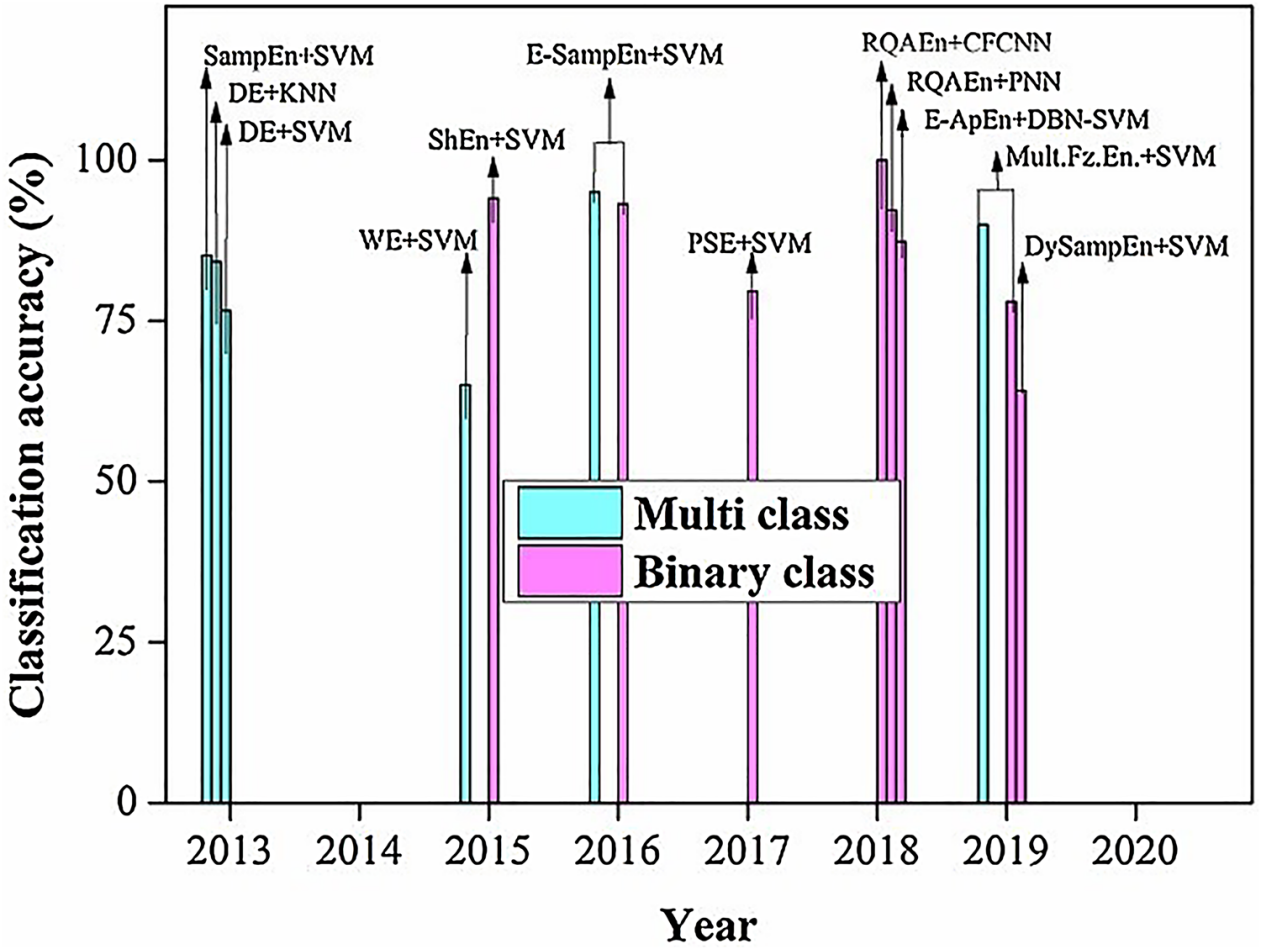
Classification accuracy of different methods with their publication year

Vijayan et al. have formulated an emotion recognition strategy that takes Shannon’s entropy as an attribute. Elucidated algorithm with a multiscale SVM classifier gives 94.097% accuracy in classifying four emotions namely excitement, happiness, sadness, and hatred [3]. Duan et al. provided a feasible study on novel EEG feature differential entropy to describe the characteristic of the thoughts and emotions. In his study, he compared DE with the traditional energy spectrum feature of frequency domain. The result shows an accuracy of 84.22% with SVM classifier over 76.56% accuracy of ES [72–74]. Authors as in [33] have extracted features using E-ApEn and employed DBN-SVM classifier for four type emotion recognition named happy, calm, sad, fear. The study shows an accuracy of 87.32% with the DBN-SVM classifier. Lu et al. published an extensive study on a newer model of pattern learning positioned on dynamic entropy to empower user-independent emotion detection from the EEG signal. The result from the study reveals that the best average accuracy of 85.11% is attained for classifying negative and positive emotions [29]. Li et al. used 18 kinds of linear and nonlinear features, in which entropies namely approximate entropy, K.S entropy, permutation entropy, singular entropy, Shannon's entropy, and spectral entropy have been used in cross-subject emotion recognition. The result shows automatic feature selection gives the highest mean recognition accuracies of 59.06% on the DEAP dataset and 83.33% on the SEED dataset [4]. Zhang et al. developed a human emotion recognition algorithm by combining EMD and sample entropy with the SVM classifier. The experiment has been done to classify four categories of emotional states such as high arousal high valence (HAHV), low arousal high valence (LAHV), low arousal low valence (LALV), high arousal low valence (HALV) on DEAP Database. The result shows the average accuracy of the proposed method is 94.98% for a binary-class task and the best accuracy achieves 93.20% for multiclass tasks, respectively, [74]. Candra et al. employed the wavelet entropy feature to build an automated EEG classifier means for human emotion recognition. Study shows that accuracy can be improved further using this method for shorter time segments and the highest accuracy of 65% for both arousal and valence emotions were achieved [51]. Lotfalinezhad separated two and three levels of emotion in arousal and valence space using a multiscale fuzzy entropy feature. Work used the DEAP database and SVM classifier and achieved a classification accuracy of 90.81% and 90.53% in two-level emotion classification and 79.83% and 77.80% in three-level classifications in arousal and valence space, respectively, for subject-dependent systems [71]. Tong et al. computed power spectral entropy and correlation dimension feature along with SVM classifier to differentiate three categories of emotion namely positive neutral and negative. Result shows with the proposed method three kinds of emotions can be classified with relatively high accuracy of 79.58% [50]. Yong et al. presented a study on emotion recognition using recurrence quantification analysis entropy with channel frequency convolutional neural network (CFCNN) as a classifier. Result gives the best accuracy of 92.24 ± 2.11% for three types of emotion recognition and also shows its remarkable efficiency over traditional methods like PSD + SVM and DE + SVM [74]. Xiang et al. in his research applied the sample entropy feature along with the SVM classifier to distinguish positive and negative emotions from EEG and achieved an accuracy of 80.43% [77]. Goshvarpour et al. in their study of emotion recognition from EEG applied several measures of recurrence quantification analysis including recurrence rate, deterministic, average line length of diagonal lines, entropy, laminarity, and trapping time along with neural network and probabilistic neural networks (PNN). The result suggests RAQ gives the best accuracy of 99.96% of three-level emotion classifications with the PNN classifier and so it can be used as an appropriate tool for emotion recognition [76]. Figure 3 graphically represents the classification accuracy of different approaches mentioned above with their publication year. We have also summarized the investigations carried out on EEG signal and entropy feature extraction in human emotion recognition in Table 1.

**Table 1:** Summary of studies conducted on EEG-based emotion recognition using entropy as a feature

Reference	No. of subjects	Emotions	Features	Database	Classifier	Accuracy
[30]	3 men, 3 women	Positive, negative	ES, DE, DASM, RASM	Private	SVM KNN	76.56% 84.22%
[77]	–	Positive, negative	Sample entropy	–	SVM weight classifier	85.11%
[51]	16 men, 16 women	Arousal, valence	Wavelet entropy	DEAP	SVM	65%
[29]	7 men, 8 women	Positive, neutral, negative	Dynamic sample entropy	SEED	SVM	64.15%
[50]	6 men, 7 women	Positive, neutral, negative	Power spectral entropy, correlation dimension	Private	SVM	79.58% 82.58%
[76]	5 men	Happy, neutral, disgust	RAQA; Shannon’s entropy and 5 others	eNTERFACE06_EMOBRAIN	Multilayer perception	36%
					Time-delay neural network	36%
					Probabilistic neural network	99.96%
[75]	5	Happy, sadness, fear	RAQA; entropy and 5 others	Private	SVM	92.24%
[3]	16 men, 16 women	Excitement, happiness, sadness, hatred	Shannon’s entropy and 3 others	DEAP	Multiclass SVM	94.097%
[33]	5 men, 5 women	Happy, calm, sad, fear	EMD approximate entropy	Private	Integration of deep belief network and SVM (DBN-SVM)	87.32%
[4]	16 men, 16 women	Excitement, happy, sadness, hatred	Approximate entropy, K-S entropy, permutation entropy, singular entropy, Shannon’s entropy	DEAP	SVM	59.8%
	7 men, 8 women	Positive, neutral, negative	Spectral entropy and 12 other nonlinear entropy methods	SEED		83.33%
[71]	16 men, 16 women	2 and 3 level of labeling in arousal and valence space	Multiscale fuzzy entropy	DEAP	SVM	2-class 90.81% (A) 90.53% (V) 3-class 79.83% (A) 77.80% (V)
[74]	16 men, 16 women	HAHV, HALV, LAHV, LALV	EMD Sample entropy	DEAP	SVM	94.98% (binary class) 93.20% (multiclass)

## Conclusions

Emotions reflect one’s mental state and can be analyzed through physiological data of the brain such as the EEG. EEG signals are dynamic and also have greater inter and intra-observer variability therefore proved to be useful in automated human emotion recognition. Features like entropy can be used to describe the chaotic nature of EEG signals as entropy the degree of variability and complexity of any system. In this paper, we have presented a comprehensive review of the use of different entropy features for recognizing human emotions using EEG signals. It should be acknowledged that the identification of real-time emotions is still in its initial phase of evolution. Because, emotions are unique to each person, the provision of a standardized method for categorizing various primary emotions remains a challenge. The majority of the framework created to date is subject dependent, and subject-independent methods need more precision. The emotion changes in the EEG signal can be observed for a very short period of about 3–15 s. Therefore, extracting the data within the subject at the moment of emotional elicitation will produce better result which requires a window-based framework in EEG processing for emotion recognition. Work can be further extended for different purposes like optimization of the above algorithm and development of a unified algorithm. There is a need for a larger-scale and well-balanced data set to avoid bias and over-fitting of the classification task. Human emotions are found to be predominantly allocated in frontal and temporal lobes, and the gamma-band is ideally suited for identifying emotions. Evidently, not all electrode locations are useful for the identification of emotion. Further studies are needed for automatic choice of selecting the suitable and optimal EEG electrode placement to enhance the efficiency and reduce the confusion of muscle signal. Added, issues such as choosing more efficient EEG emotional attributes and reducing the interference from the exterior surrounding are required to be studied. Sturdier and progressive feature extraction techniques such as machine learning should be considered. Emotion experiences can differ in both individuals male and female, so developing a cross-gender EEG emotion detection framework can be a crucial problem to be investigated for emotion identification to be more generalized. Some research shows that correlation analysis is not really adequate to predict the purpose of certain functionalities as well as to determine the places of relevance. In forthcoming researches, more investigation from multiple points of view employing different methods will be expected. The use of the smallest number of EEG channels can probably boost user comfort and cut down the related computation cost. Another potential research work could be to develop a framework based on these features for emotion-related to mental disorder identification such as depression.

Further studies can also be done on the influence of other unused entropy features for emotion recognition. Also, there is still a need to reduce the calculation cost of used entropies. EEG does have a very high temporal resolution but a relatively lower spatial resolution. So, the precise classification can be gained by integrating EEG with some higher spatial resolution signals such as NIRS and fMRI.

**Fig. 4:**
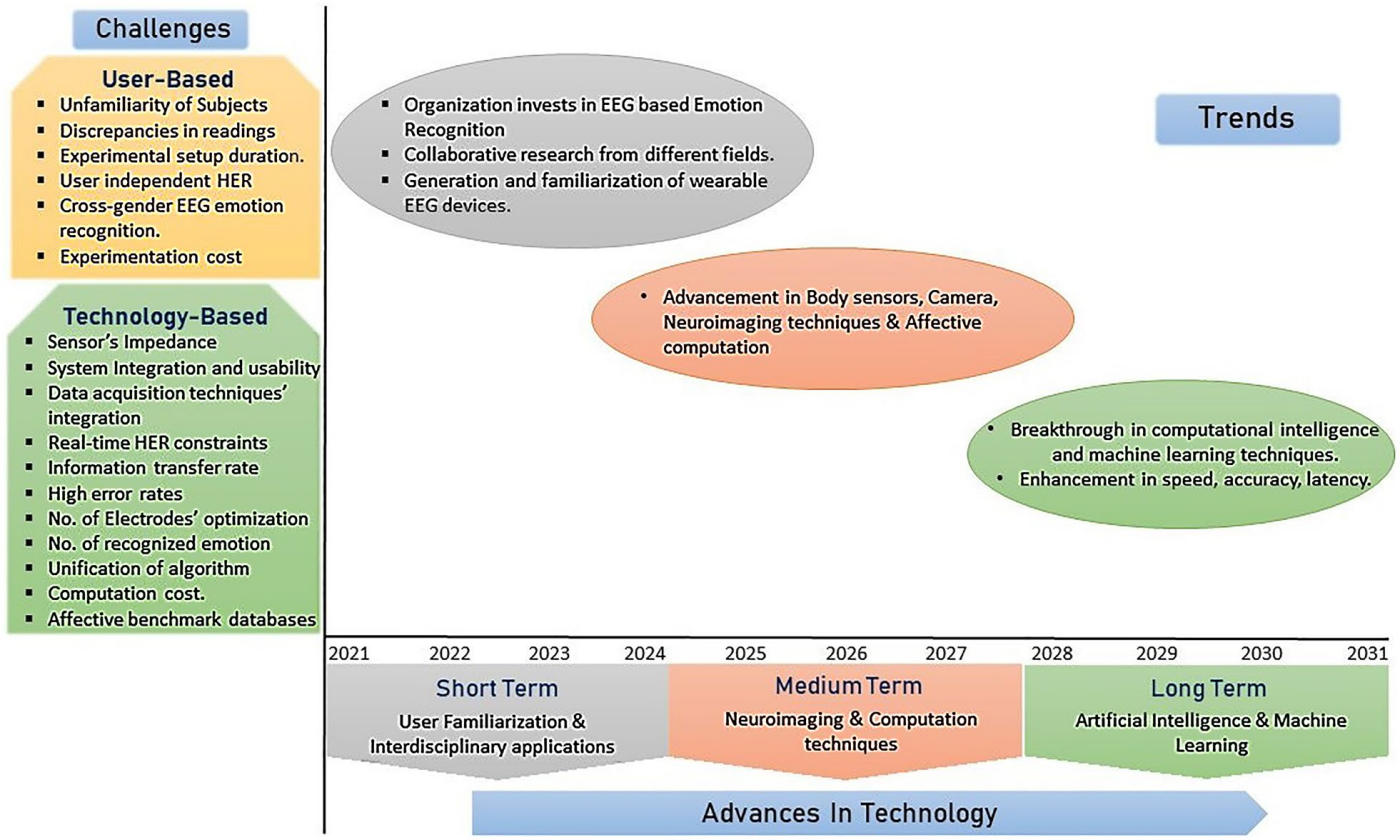
Problems identified and possible advancement in the EEG-based emotion recognition research

There are in total 8 valence levels of emotion. Most of the researches done to date is rough emotion classification, i.e., it only classifies a maximum of three or four types of emotion. Future research must focus on the classification of more detailed or all eight types of valence levels. Figure 4 summarizes the paper, presenting direction for future study, issues, and developments in EEG-based human emotion recognition from our viewpoint. It illustrates several issues associated with emotion recognition in BCI framework, which in general is divided into two domains: technology based and user based. It also shows how the advances in technology and trends would affect the field of EEG-based human emotion recognition research. Emotion identification in the field of BCI continues to be challenging and needs more research and experimentation. Higher research on designing a reliable and emotion classification system is still a needful job to implement a seamless communication between humans and machines.

## Data Availability

Not applicable.

## Abbreviations

EEG: Electroencephalography
BCI: Brain–computer interface
IAPS: International affective picture system
DEAP: Dataset for emotion analysis using physiological signals
SEED: SJTU Emotion EEG Dataset
SampEn: Sample entropy
DySampEn: Dynamic sample entropy
DE: Differential entropy
PSE: Power spectral entropy
WE: Wavelet entropy
EMD ApEn: Empirical Mode Decomposition Approximate Entropy
KSE: Kolmogorov Sinai entropy
PE: Permutation entropy
RQAEn: Recurrence quantification analysis entropy
KNN: K-Nearest Neighbor
SVM: Support Vector Machines
DBN-SVM: Deep Belief Network-Support Vector Machines
CFCNN: Channel frequency convolutional neural network
PNN: Probabilistic neural network
HAHV: High arousal high valence
HALV: High arousal low valence
LAHV: Low arousal high valence
LALV: Low arousal low valence
fMRI: Functional Magnetic Resonance Imaging
NIRS: Near infrared spectroscopy
